# Dioscin inhibiting EGFR-mediated Survivin expression promotes apoptosis in oral squamous cell carcinoma cells

**DOI:** 10.7150/jca.85011

**Published:** 2023-07-09

**Authors:** Ming Li, Qin Zhao, Jinzhuang Liao, Xiaocong Wang, Lulu Liu, Xiaoyue Zhang, Lijun Liu, Haidan Liu, Shusheng Zhang

**Affiliations:** 1 Changsha Stomatological Hospital, Changsha, Hunan 410004, China.; 2 School of Stomatology, Hunan University of Chinese Medicine, Changsha, Hunan 410208, China.; 3 Department of Radiology, The Third Xiangya Hospital of Central South University, Changsha, Hunan 410013, China.; 4 Clinical Center for Gene Diagnosis and Therapy, The Second Xiangya Hospital of Central South University, Changsha, Hunan 410011, China.

**Keywords:** Dioscin, EGFR, survivin, oral squamous cell carcinoma, apoptosis

## Abstract

Overexpression of survivin plays a crucial role in tumorigenesis and correlates with poor prognosis in human malignancies, including oral squamous cell carcinoma (OSCC). Thus, survivin has been proposed as an attractive target for new antitumor interventions. In the present study, we found that a natural compound, Dioscin, inhibited OSCC cells by reducing the survivin protein level and activating apoptotic signaling. Dioscin inhibits survivin expression by interrupting EGFR binding to the AT-rich sequences (ATRSs) at the survivin promoter, eventually promoting survivin-mediated cell apoptosis. The *in vivo* study showed that Dioscin suppressed the tumor development of SCC25 cells. Furthermore, the immunohistochemistry (IHC) results revealed that treated with Dioscin reduced the protein levels of EGFR and survivin in SCC25 xenograft tumors. Overall, our findings indicate that targeting the EGFR-survivin axis might be a promising OSCC treatment strategy.

## Introduction

Oral squamous cell carcinoma (OSCC) is the most common subtype of head and neck squamous cell carcinoma (HNSCC) 1 and represents about 90% of all oral malignancies 2. Tobacco, alcohol, and betel quid are the most known carcinogens contributing to the high occurrence of OSCC 3. Other factors, such as human papillomavirus (HPV) infection, may also be involved 4. Despite technological advances in diagnosis and treatment strategies, such as chemotherapy, radiotherapy, target therapy, photodynamic therapy and surgery, the prognosis of OSCC has only moderately improved and the 5-year survival rate of patients with OSCC remains approximately 50% 5, 6. The discovery of more effective agents with fewer side effects may provide new avenues for treating OSCC.

Many natural products from medicinal and edible plants have anticancer activity, low toxicity, and few adverse side effects making them a source of prototype molecules to develop new chemotherapeutic agents with potent anticancer properties 7-11. One of these compounds is dioscin, a steroidal saponin, which is abundantly expressed in many medicinal plants, such as *Dioscorea nipponica Makino*. Numerous research has suggested that dioscin has broad-spectrum pharmacological activities, such as anti-oxidative, anti-fibrosis, anti-proliferation, anti-allergic, anti-viral, anti-inflammation, anti-fungal, immunoregulation, anti-hyperuricemia, anti-aging, pro-autophagic, anti-thrombotic, and cholesterol-lowering effects and possesses fewer side effects compared with the drugs with the same effect in current clinical application relatively speaking 12-14. Dioscin also provided obvious protective effects on various metabolic disorders, such as fatty acid metabolism and the damage of organs, including the kidney, liver, lung, and heart 15-17. Furthermore, dioscin reduced chemoresistance 18 and regulated M1 macrophage polarization and differentiation of myeloid-derived suppressor cells 19. Moreover, dioscin has shown its antitumor activity in various types of cancer, including lung 20, colorectal 12, prostate 21, gastric 22, ovarian 23, and laryngeal 24 cancers through JNK, MAPK, and Akt/mTOR signaling 20, 23. However, the effect of dioscin activity on human OSCC cells has not been reported, and the underlying mechanism remains unknown to our best knowledge.

Increasing evidence indicates that survivin (encoded by the gene *BRIC5*), a member of the inhibitor of apoptosis (IAP) protein family, is frequently overexpressed 25 and generally associated with a more aggressive disease progression, poor clinical outcomes and therapeutic resistance in a wide variety of human cancers 26. Aberrant survivin expression has been found in lung cancer 27, hepatocellular carcinoma 28, breast cancer 29, and oral squamous cell carcinoma 26. Survivin inhibits apoptosis of tumor cells by regulating caspase 3/7 or apoptosis-regulatory factors such as HSP90 and AIF 30. It also can promote mitosis or DNA repair via Ku70 30. Thus, targeting survivin would be a plausible therapeutic method for OSCC treatment.

Therefore, this paper aimed to investigate the effects of dioscin against OSCC cells, and the underlying mechanism associated with the EGFR/Survivin pathway was also studied. The findings may provide novel insights and develop a potent candidate for preventing and treating OSCC.

## Materials and Methods

### Reagents and antibodies

Dioscin (> 98% purity, #HY-N0124) was purchased from MedChemExpress (Monmouth Junction, NJ). Other chemical reagents, such as dimethylsulfoxide (DMSO), NaCl, SDS, and Tris base for buffer preparation, were obtained from Sigma-Aldrich (St. Louis, MO). The FBS, cell culture media, and antibiotics were purchased from Invitrogen (Grand Island, NY). Antibodies against Survivin (#2808, IB:1:1000), EGFR (#4276, IB: 1:1000), cleaved-caspase 3 (#29664, IB: 1:1000), cleaved-PARP (#5625, IB: 1:1000), CREB (#9197, IB: 1:1000), anti-rabbit IgG HRP (#7074), and anti-mouse IgG HRP (#7076) were obtained from Cell Signaling Technology, Inc. (Beverly, MA). β-actin (#A5316, IB: 1:10000) was purchased from Sigma-Aldrich (St. Louis, MO, USA). Dioscin was dissolved in DMSO at a stock solution concentration of 100 mM. The stock solution was freshly diluted to desired concentrations for each experiment.

### Cell lines and cell culture

Human oral squamous cell carcinoma cell lines SCC-4 (ATCC CRL-1624) and SCC-25 (ATCC CRL-1628) were purchased from the American Type Culture Collection (ATCC, Manassas, VA). All cells were cultured with DMEM/F12 medium containing 10% of FBS and 1% antibiotics and maintained at 37˚C in a humidified incubator with 5% CO_2_ according to the ATCC protocols. The cells were cytogenetically tested and authenticated before being frozen.

### Protein preparation and Western blotting

Whole-cell extract (WCE) was prepared with RIPA buffer (20 mM NAP, pH 7.4, 150 mM NaCl, 1% Triton, 0.5% Sodium-deoxycholate, and 0.1% SDS) supplemented with protease inhibitors. BCA assay (#23228, Pierce, Rockford, IL) was used for protein concentration following the standard procedures. Western blotting was performed as previously described 31, 32. Briefly, WCE was boiled with loading buffer at 95°C for 5 min and subjected to SDS-PAGE followed by electrotransfer to the PVDF membrane. The membrane was blocked with 5% non-fat milk and incubated with primary antibody at 4°C overnight. Anti-rabbit IgG HRP and anti-mouse IgG HRP were used as second antibodies. The target protein was visualized using the ECL substrate (#32106, Thermo Fisher Scientific).

### Cell viability assays

Human OSCC cells were seeded at a density of 2×10^3^/well in 96-well plates in 100 μL of DMEM/F12 medium containing 10% of FBS without or with different concentrations of dioscin and incubated in a 37°C, 5% of CO_2_ incubator. After culturing for 48 h, 10 μL of the WST-1 reagent (#11644807001; Roche, Mannheim, Germany) were added to each well, and cells were incubated for 2 hours at 37°C. The absorbance of the cellular reduction of WST-1 to formazan was measured at 450 nm as previously described 33. Three independent experiments were performed in triplicate.

### Flow cytometry

Flow cytometry was performed as previously described 34. OSCC cells were seeded into 6-well plates in DMEM/F12 medium containing 10% of FBS. After culturing for 12 h, different concentrations of dioscin were added to each well and left on the cells for 48 h. After treatment, attached and floating cells were harvested. For apoptosis analysis, the cells were suspended in 1×10^6^ cells/mL, and 5 μL Annexin V and Propidium Iodide staining solution were added to 300 μL of the cell suspension. After incubated 10-15 minutes at room temperature in the dark, stained cells were assayed and quantified using a FACSort Flow Cytometer (BD, San Jose, CA, USA). Each experiment was done in triplicate and repeated at least twice.

### Transfection and luciferase reporter assays

The EGFR WT expression construct (#11011) and the empty vector (#1764) were available on Addgene (Cambridge, MA, USA). The *pGL3-survivin promoter* plasmid was kindly provided by Prof. Ning-Zhi Xu at the Chinese Academy of Medical Sciences 35. The *pGL3-Basic* and the Renilla luciferase reporter construct *pRLSV40* (Promega, Madison, WI, USA) was used as previously described 36. Human OSCC cells growing on 24-well plates were transfected with the *pGL3-survivin promoter* plasmid or the *pGL3-Basic* vector. After transfection, cells were treated with 0.1% DMSO or dioscin for 48 h. Or OSCC cells were co-transfected with the *pGL3-survivin promoter* plasmid or the *pGL3-Basic* vector along with EGFR WT or empty vector for 48 h using Lipofectamine 2000 (cat#11668-019, Invitrogen, Carlsbad, CA) following the manufacturer's instructions. Each transfection contained the *pRL-SV40* construct. Firefly luciferase and *Renilla* luciferase activity were determined using the Dual-Luciferase reporter assay system (#E1910, Promega) with a GloMax 20/20 luminometer (#E5311, Promega). Relative luciferase activities were normalized for transfection efficiency by dividing Firefly luciferase values by *Renilla* luciferase values. The data are represented as the fold induction compared to the *pGL3-Basic* vector. All experiments were performed in triplicate with three independent experiments. The overexpression of EGFR protein was verified by Western blotting analysis.

### Chromatin-immunoprecipitation assay

Chromatin-immunoprecipitation (ChIP) assays were performed as previously described 36. Briefly, the dioscin-treated OSCC cells were cross-linked with 1% of formaldehyde, neutralized with 125 mM glycine, harvested, and disrupted by sonication to fragments with an average size of ~500 bp. The chromatin of cells was pre-cleared with 30 μL protein G agarose/salmon sperm DNA (#16-201; Upstate, Temecula, CA, USA) and incubated with 2 μg of EGFR (#MA5-13697; ThermoFisher), or normal mouse IgG (#12-371; Millipore) antibody at 4°C overnight. The immunocomplexes were pulled down with 30 μL dynabeads Protein G (#100.03D; Invitrogen, Carlsbad, CA, USA). The beads were collected on a magnetic device and washed with ChIP wash buffer and TE buffer (10 mM Tris-HCl, pH 8.0, 1 mM EDTA). Cross-links for both ChIP and input DNA were reversed at 65°C for 5 h and DNA was purified with E.Z.N.A Cyclepure Kit (Omega BIO-TEK, Norcross, GA, USA). Equal amount of each ChIP-DNA was used as a template for polymerase chain reactions (PCR). PCR products were analyzed by electrophoresis on a 3% agarose gel and visualized by ethidium bromide staining. The primer pairs** (Table 1)** were used to amplify the* survivin* promoter regions in the immunoprecipitated DNA.

### *In vivo* tumor growth

All animal experiments were approved by the Institutional Animal Care and Use Committee (IACUC) of Hunan University of Chinese Medicine (Changsha, China). The OSCC xenograft models were constructed by s.c.injection of SCC-25 (3×10^6^) cells into the right flank of 6-week-old athymic nude mice (n=5). Tumor volume and mouse body weight were recorded every two days. The tumor-bearing mice were initiated with compound treatment when the tumor volume reached around 100 mm^3^. The control group was administered vehicle control, whereas the compound-treated group was administered Dioscin (5 mg/kg) every two days by i.p. injection 37, 38. Tumor volume was determined using the formula length × width × width/2. The tumor mass was fixed and subjected to Immunohistochemical staining.

### Immunohistochemical staining

Immunohistochemical staining was performed as previously described 39. The mice xenograft tumor tissue slides were deparaffinized and rehydrated by subsequent incubation with xylene and ethanol to complete paraffin removal. Antigen retrieval was performed by submerging the tissue slides into sodium citrate buffer (10 mM, pH 6.0) and boiling for 10 min. After a wash with ddH_2_O 3 times, the slides were incubated with 3% H_2_O_2_ in methanol for 10 min to deactivate the endogenous horseradish peroxidase, followed by washing with PBS 3 times. The slides were blocked with 50% goat serum albumin in PBS at room temperature for 1 h and hybridized with the primary antibody in a humidified chamber overnight at 4 °C. Tissue slides were incubated with secondary antibody at room temperature for 45 min and visualized by DAB substrate. Hematoxylin was used for counterstaining.

### Statistical analysis

Statistical analyses were performed using GraphPad Prism 9 (GraphPad 9, San Diego, CA, USA). The quantitative data are expressed as mean ± SD. The difference was evaluated using the Student's *t*-test or ANOVA. A probability value of *p*< 0.05 was used as the criterion for statistical significance.

## Results

### Dioscin inhibits OSCC cell growth

**Figure 1:**
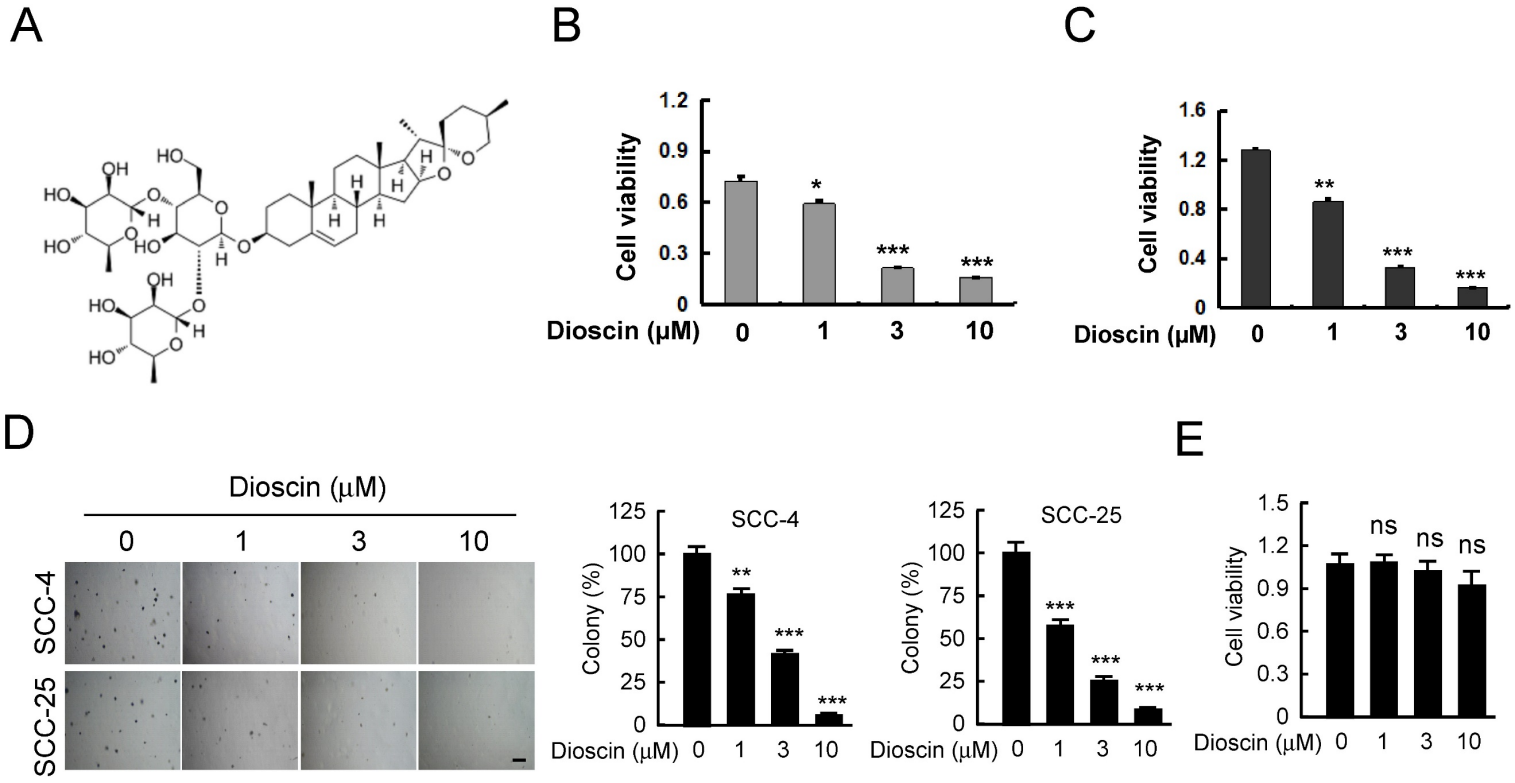
**Dioscin inhibits cell viability of OSCC cells. (A)** Dioscin chemical structure.** (B, C)** Dioscin suppresses cell viability of SCC-4** (B)** and SCC-25**(C)** OSCC cells. SCC-4 and SCC-25 OSCC cells were treated with DMSO or the indicated concentrations of dioscin in medium containing 10% FBS, and growth was measured at the indicated times using the WST-1 assay. Data represent mean ± SD from three independent experiments. *, *p*<0.05, **, *p*<0.01, ***, *p*<0.001, a significant difference from the DMSO control cells.** (D)** Dioscin inhibits the colony formation of SCC-4 and SCC-25 cells in soft agar. **(E)** The effect of Dioscin on HaCat cells was determined by WST-1 assay. ns, not statistically significant.

Dioscin (Fig. 1A) has shown antitumor activities against several human cancers. In order to identify the antitumor effect of dioscin on OSCC, we detected the suppression effect of dioscin on cell proliferation in SCC-4 and SCC-25 cells. WST-1 results indicated that treatment with dioscin dose-dependently suppressed cell proliferation in both SCC-4 (Fig. 1B) and SCC-25 cells (Fig. 1C). Moreover, the soft agar assay showed that treatment with dioscin inhibited the colony formation of SCC-4 and SCC-25 cell significantly (Fig. 1D). To test the toxicity of dioscin to normal cells, we treated the immortalized epithelial cell HaCat with dioscin, and found that dioscin could not significantly reduce cell viability at 10 µM (Fig. 1E). These results suggest that dioscin specifically suppresses the growth of OSCC cells.

### Dioscin induces cell apoptosis in OSCC cells

**Figure 2:**
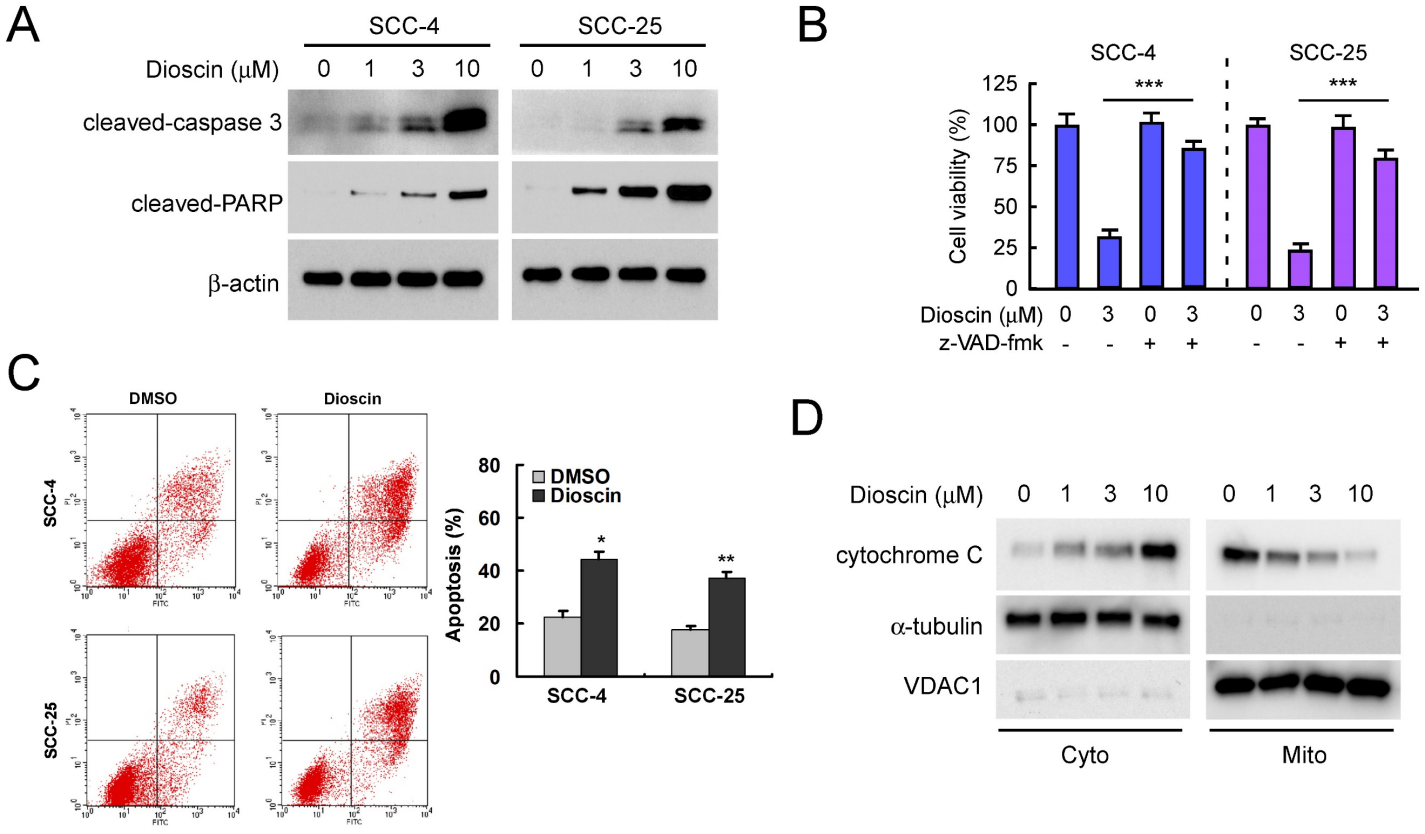
** Dioscin induces apoptosis of OSCC cells. (A)** SCC-4 and SCC-25 cells were treated with DMSO or the indicated concentrations of dioscin for 48 h, and whole cell extracts were analyzed by Western blotting. Cleavage of caspase 3 and PARP were detected after dioscin treatment. β-actin was used as a loading control. **(B)** SCC-4 and SCC-25 cells were treated with DMSO or the indicated concentrations of dioscin for 24 h, then pan-caspase inhibitor z-VAD-fmk was added to the cell culture medium and maintained for another 24 h. Cell viability was examined by WST-1 assay. **(C)** SCC-4 and SCC-25 cells were treated with DMSO or dioscin (3 µM) for 48 h, stained by propidium iodide and Annexin V-FITC, then analyzed by flow cytometry to determine the apoptotic cells. Data represent mean ± SD from two independent experiments. *, *p<*0.05, **, *p<*0.01, a significant difference compared with the DMSO-treated cells. **(D)** SCC-4 cells were treated with DMSO or the indicated concentrations of dioscin for 48 h, and subcellular fractions were isolated and subjected to Western blotting analysis.

We next tested the effect of dioscin on cell death in SCC-4 and SCC-25 cells. Western blotting results showed that treatment with dioscin dose-dependently resulted in enhanced cleavage of procaspase 3 and PARP in both SCC-4 and SCC-25 cells (Fig. 2A). Treated with pan-caspase inhibitor, z-VAD-fmk, restored cell viability in both SCC-4 and SCC-25 cells (Fig. 2B). Furthermore, cell death was assessed by flow cytometry analysis upon Annexin V/PI staining using SCC-4 and SCC-25 cells as models. The results demonstrated that the application of different concentrations of dioscin for 48 h dose-dependently triggered significant apoptosis (Fig. 2C). The subcellular fractions assay revealed that dioscin-induced the release of cytochrome C from mitochondrial to the cytoplasm (Fig. 2D). These results suggest that dioscin-mediated OSCC cell growth inhibition may partly due to dioscin-induced intrinsic apoptosis.

### Dioscin suppresses EGFR-regulated survivin expression in OSCC cells

**Figure 3:**
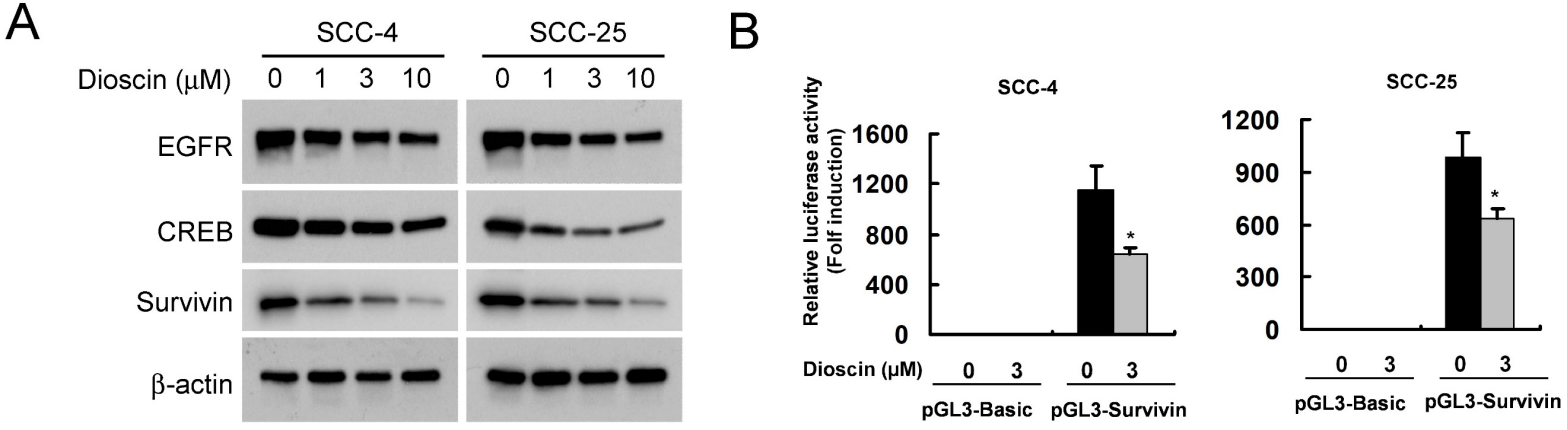
** Effect of dioscin on the expression Survivin in OSCC cells. (A)** SCC-4 and SCC-25 cells were treated with DMSO or the indicated concentrations of dioscin in medium containing 10% FBS for 48 h. After treatment, attached and floating cells were harvested. Expression of the indicated proteins was analyzed by Western blotting with specific antibodies. β-actin was used as a loading control. **(B)** Dioscin inhibits the Survivin promoter activity in OSCC cells. Dual luciferase reporter assays of plasmid DNA encoding a fragment of human Survivin promoter in OSCC cells were performed as described in Materials and Methods. SCC-4 and SCC-25 cells were transfected with the Survivin promoter reporter plasmid or pGL3-Basic vector and then exposed to DMSO or dioscin (3 µM) for 48 h. Firefly luciferase readings were normalized to Renilla luciferase to correct for transfection efficiency. The Survivin promoter-driven luciferase activities were expressed as fold induction over the activity of the pGL3-Basic vector. Data represent mean ± SD from two independent experiments performed in triplicate. *, *p*<0.05, significant difference compared with the DMSO control cells.

**Figure 4:**
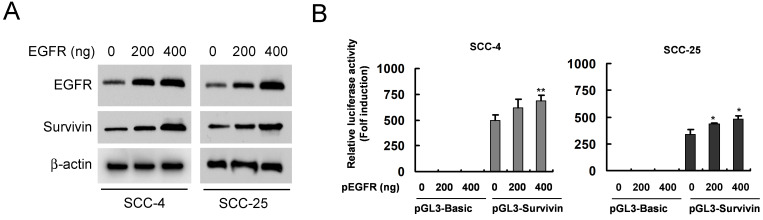
** Effect of EGFR on the expression Survivin in OSCC cells. (A**) Overexpression of EGFR was examined by Western blot analysis with specific antibodies. β-actin was used as a loading control. (**B**) Overexpression of EGFR increases the Survivin promoter activity in OSCC cells. SCC-4 (left panel) and SCC-25 (right panel) cells were co-transfected pGL3-Survivin plasmid or pGL3-Basic vector along with EGFR WT expression construct (#11011) or empty vector for 48 h as described in Materials and Methods. The Survivin promoter activity was measured by dual luciferase reporter assays. Firefly luciferase readings were normalized to Renilla luciferase to correct for transfection efficiency. The Survivin promoter-driven luciferase activities were expressed as fold induction over the activity of the pGL3-Basic vector. Data represent mean ± SD from two independent experiments performed in triplicate. *, *p*<0.05, **, *p*<0.01, a significant difference compared with the empty vector-transfected control cells.

We found that dioscin downregulated the survivin protein level (Fig. 3A). Based on the finding that survivin is highly expressed in OSCC tissues and cell lines 7, we thus examined the impact of dioscin on the expression of the survivin gene in OSCC cells by luciferase reporter gene assay. The expression of luciferase in the survivin-Luc reporter was driven by the survivin promoter. Dioscin treatment decreased luciferase expression of the reporter plasmid in SCC-4 and SCC-25 cells (Fig. 3B), indicating that dioscin inhibited the expression of the survivin gene in OSCC cells. Notably, we found that suppression of survivin by dioscin was accompanied by downregulation of EGFR protein (Fig. 3A). To further determine whether EGFR involves in the activation of the survivin promoter, the pGL3-Survivin-Luc reporter was co-transfected with the EGFR expression plasmid to assess the contribution of EGFR to the survivin promoter activity. Results demonstrated that overexpression of EGFR (Fig. 4A) significantly increased the protein level of Survivin and the promoter activity of survivin in both SCC-4 and SCC-25 cells (Fig. 4B). Collectively, the data suggest that dioscin suppresses EGFR-regulated survivin expression in OSCC cells.

### Dioscin inhibits the direct binding of EGFR to the survivin gene locus in OSCC cells

**Figure 5:**
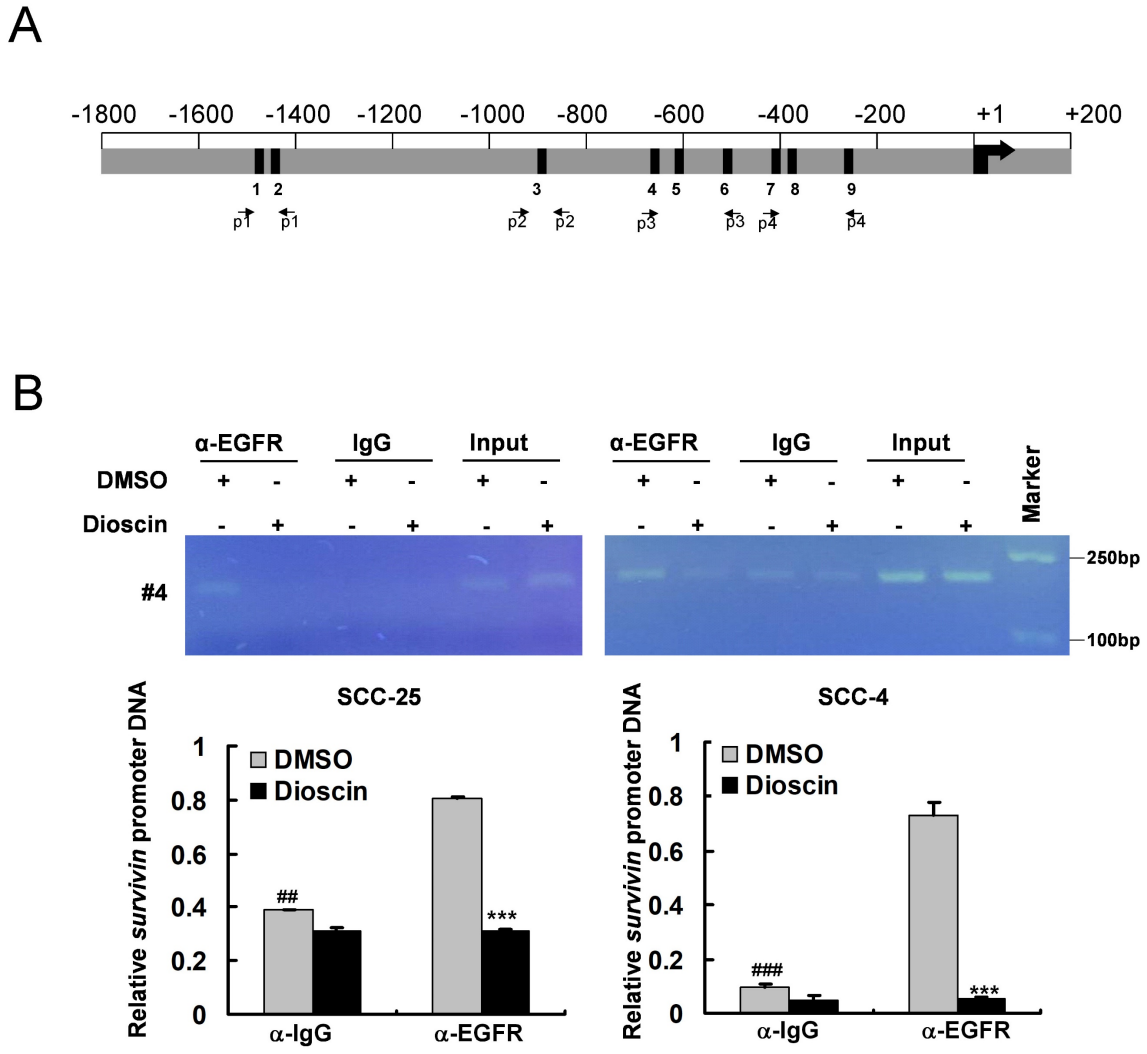
** Dioscin inhibits the binding of EGFR to the *Survivin* promoter in OSCC cells. (A)** Nine predicted EGFR targeting sites (the ATRS1-9) in the Survivin promoter. This graph shows the Survivin promoter region from -1800 to +200. The black bars mean the putative ATRSs. ATRS-1: -1497 to -1493; ATRS-2: -1473 to -1469; ATRS-3: -892 to -888; ATRS-4: -639 to -635; ATRS-5: -608 to -604; ATRS-6: -557 to -553; ATRS-7: -403 to -399; ATRS-8: -395 to -391; ATRS-9: -261 to -257. The location of primers (#1 to #4) used in ChIP experiments is indicated.** (B)** SCC-4 and SCC-25 cells were treated with DMSO or dioscin (3 µM) for 48 h and subjected to ChIP assays with an antibody against EGFR or normal mouse IgG. The precipitated DNA fragments were subjected to PCR analysis to test for the presence of sequences corresponding to the Survivin gene locus. Input material (10%) was shown for comparison. ##, *p*<0.01, ###, *p*<0.001, significant difference compared with the IgG control. ***, *p*<0.001, a significant difference compared with the DMSO control cells.

It has been reported that EGFR can be activated and then translocated into the nucleus as a transcriptional activator 40, 41. The putative EGFR-targeting sites are AT-rich sequences (ATRSs), including TNTTT or TTTNT 40, 41**.** Sequence analysis revealed nine putative ATRSs at the survivin promoter (between -1497 and -257, ATRS1-9, Fig. 5A). To verify whether EGFR can bind to the survivin promoter, we performed an *in vivo* ChIP assay. Further, to demonstrate the effect of dioscin on the interaction of EGFR with the survivin promoter *in vivo*, OSCC cells were treated with DMSO or dioscin for 48 h, chromatin was immunoprecipitated, and the binding of EGFR to the AT-rich sequences was analyzed by PCR using primers designed around the survivin promoter region (Fig. 5A and Table 1). ChIP assays with SCC-4 and SCC-25 cells showed that EGFR interacted with the survivin promoter region (Fig. 5B) and dioscin significantly disrupted the interaction of EGFR with the survivin locus (Fig. 5B). These results suggest that dioscin inhibits the direct binding of EGFR to the survivin promoter to decrease survivin expression, leading to dioscin-induced apoptosis in OSCC cells.

### Dioscin suppresses the *in vivo* tumor growth of SCC-25 cells

**Figure 6:**
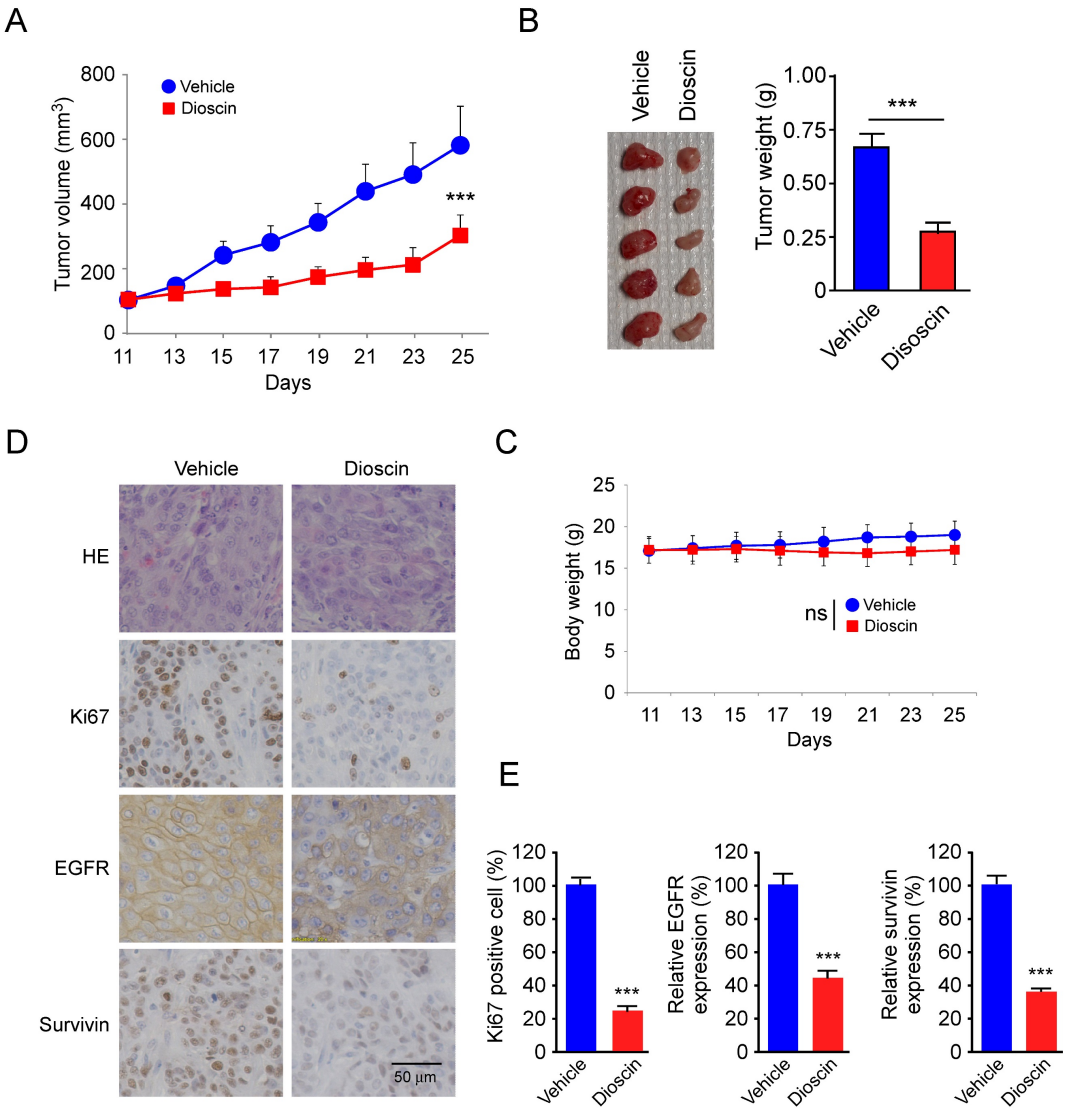
** Dioscin inhibits the *in vivo* tumor growth of SCC-25 cells. (A, B)** The tumor volume **(A)**, the image of the tumor mass and, weight** (B)** of SCC-25-derived xenograft tumors treated with vehicle or dioscin. **(C)** The body weight of SCC-25-derived xenograft tumors treated with vehicle or dioscin.** (D, E)** The representative images **(D)** and qualifications** (E)** of IHC staining of Ki67, EGFR, and survivin in SCC-25-derived xenograft tumors with vehicle or dioscin treatment. ***p<0.001. Scale bar, 50 μm.

To further determine the *in vivo* antitumor effect of dioscin, we performed a xenograft mouse model using SCC-25 cells. The results showed that the tumor volume of the vehicle-treated group of SCC-25-derived tumors was 574 ± 121 mm^3^. In contrast, dioscin treatment dramatically suppressed tumor development, as the tumor volume was only 294 ± 64 mm^3^ (Fig. 6A). We next examined the tumor weight of both vehicle- and dioscin-treated groups and found that dioscin reduced tumor weight by over 50% compared to that of the vehicle-treated group (Fig. 6B). In addition, we found that treated with dioscin did not reduce body weight significantly (Fig. 6C), indicating that dioscin was well tolerated at the current dosage. Immunohistochemistry (IHC) staining revealed that administration with dioscin reduced the expression of Ki67 in SCC-25 tumors. Also, EGFR and survivin protein levels were reduced consistently (Fig. 6D and 6E). These results suggest that dioscin inhibited the *in vivo* tumor growth of SCC-25 cells.

## Discussion

**Figure 7:**
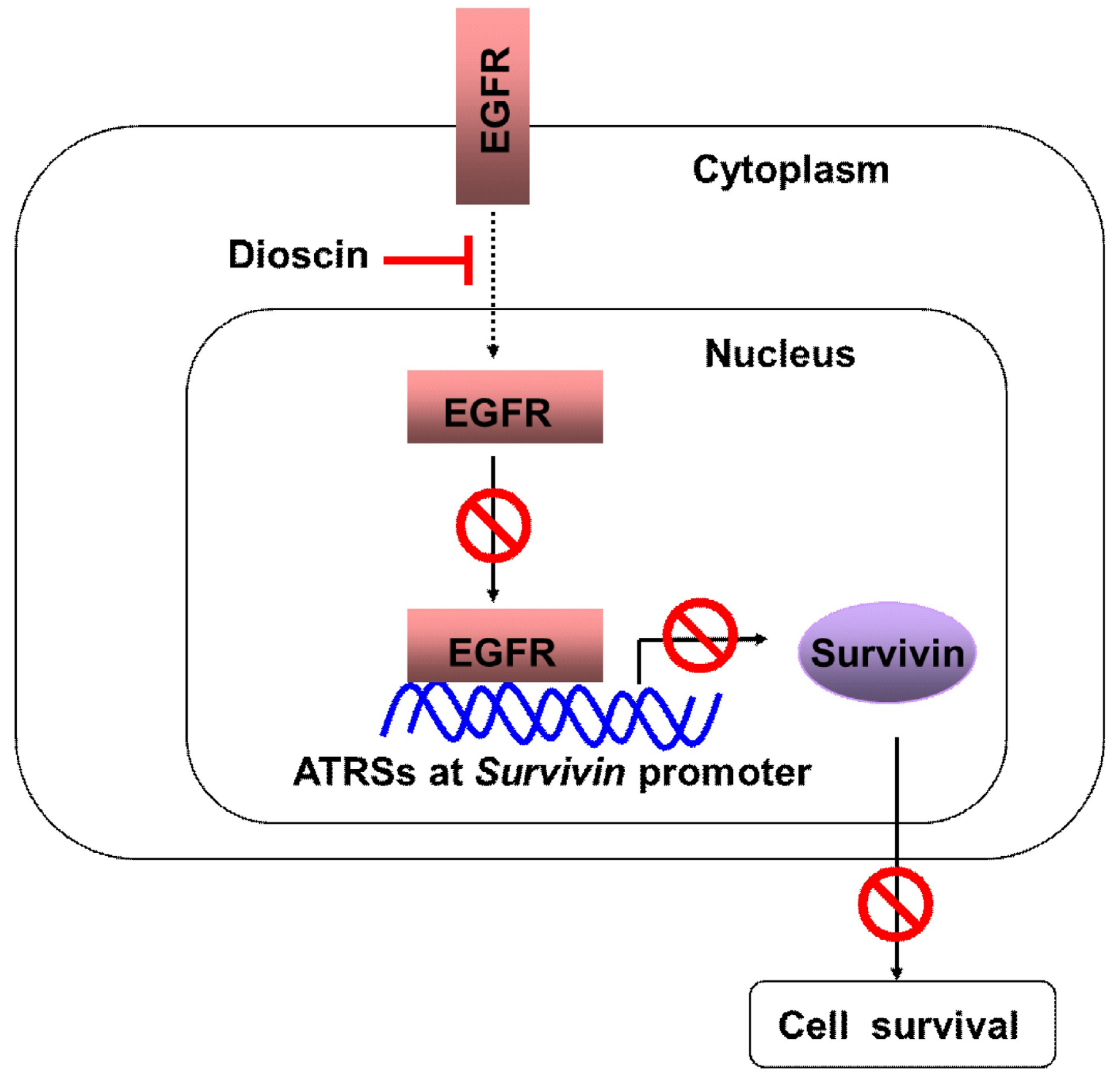
** Schematic model of the inhibition of EGFR-regulated Survivin expression by dioscin**. Dioscin inhibited EGFR binding to the Survivin promoter, which induced apoptosis through suppressing Survivin expression.

In the present study, we have shown that a natural compound, dioscin, inhibits the growth of OSCC cells. One of the major mechanisms seems to be the suppression of survivin and the promotion of cell apoptosis. Moreover, we demonstrated that the downregulation of survivin is regulated by inhibiting the direct binding of EGFR to the survivin promoter in OSCC cells (Fig. 7).

Increased levels of survivin gene expression are observed in human cancers 25. Previous studies have reported that survivin expression was higher in the cancer samples compared to leukoplakia samples and in normal tissues 42. High survival expression is correlated with lymph node metastasis, clinical stage, and poor prognosis 43, 44. Since survivin plays a crucial role in OSCC, survivin-targeted therapeutics have remained a central goal of survivin studies in the cancer field, including OSCC. Currently, strategies based on different mechanisms have developed several survivin inhibitors, including survivin-partner protein interaction inhibitors, survivin homodimerization inhibitors, survivin gene transcription inhibitors, survivin mRNA inhibitors, and survivin immunotherapy 45. Survivin has become a well-known cancer therapeutic target.

Epidermal growth factor receptor (EGFR) is a tyrosine kinase receptor located at the cell membrane. Aberrant EGFR is considered an etiological factor in human cancer, contributing to cancer development, metastasis, and resistance to chemotherapy 46. Huang et al. reported that overexpression and increased gene copy numbers of EGFR were found in oral squamous cell carcinomas (OSCC). EGFR protein overexpression is closely related to EGFR gene amplification and is associated with a poor prognosis both in terms of disease-free survival (DFS) and overall survival (OS) 47. Several other studies also suggest that EGFR overexpression is frequently observed and plays a crucial role in the pathogenesis of human oral cancer, making it a potential therapeutic target 48. Thus, EGFR tyrosine kinase inhibitors, such as Erlotinib 49, and EGFR monoclonal antibodies, such as cetuximab 50, have investigated the antitumor activity against human oral cancer.

Accumulating evidence indicated that cell surface receptors, such as EGFR family members (c-erbB-1/EGFR, c-erbB-3, and c-erbB-4) can translocate to nuclear and exert biological function 51. For example, c-erbB-3 is located in the nucleus through the active nuclear localization signal (NLS) near the COOH-terminus of the protein upon the addition of the exogenous ligand 52. EGFR can recognize AT-rich sequence sites (ATRSs) of target gene promoters and function as a transcription factor to activate genes expression 40, 41, such as cyclin D1 40, iNOS 53, COX-2 54, Aurora A 41 and MMP2 55. Cordero et al. 56 reported that 1,25(OH)_2_D_3_ inhibited cell proliferation by decreasing EGF-induced nuclear translocation and EGFR binding to ATRS sequence in the cyclin D1 promoter, finally suppressing cyclin D1 transcription. In addition, it has been reported that FGFR-1 acted as a transcription factor at the FGF-2 promoter 57. These findings suggest that direct nuclear translocation of cell surface receptors, such as EGFR, may act as a transcription factor to regulate gene expression.

Expression of the survivin gene can be regulated at the transcriptional level. Analysis of human survivin promoter regions for potential transcription factor binding sites revealed consensus sequences including Stat3 24, NF-κB 58, Rb 59, Sp1 60, p53 61, and Egr1 62, suggesting a possible involvement of these factors in the control of the survivin gene expression. In addition to being modulated at the transcriptional level by various transcription factors that bind and activate the survivin promoter, survivin could be regulated on multiple levels, such as translational and post-translational. For example, E3 ubiquitin ligase XIAP 63, CUL9 64, FBXL7 65, and deubiquitinases STAMBPL1 66, USP1 67 and USP19 68 have been identified to modulate ubiquitination of survivin. Moreover, survivin transcripts can be influenced by microRNAs (miRs). For example, miR-494 has been demonstrated to downregulate survivin expression, inhibit cell proliferation and colony formation in gastrointestinal stromal tumors 69, and promote cell apoptosis in TEL-AML1^+^ leukemia 70. Epigenetic regulation of survivin, including methylation status of the survivin promoter and histone modifications, may also contribute to survivin regulation 71. Future studies need to explore whether these mechanisms contribute to the elevated survivin protein in human OSCC.

Several small molecule inhibitors exert their anti-cancer activity by decreasing survivin gene expression. YM155 showed anti-cancer activity both *in vitro* and *in vivo* by strongly inhibiting the survivin promoter activity and survivin expression 72. One possible mechanism by which YM155 inhibits survivin expression involves the abrogation of Sp1 interacting with the Sp1 DNA-binding site at the survivin promoter 73. Small molecule FL118 is another promising anti-cancer agent inhibiting multiple cancer-associated survival and treatment-resistant proteins, including survivin, Mcl-1, XIAP, cIAP2, and MdmX 74. SF002-96-1 inhibits Colo320 cell growth by inhibiting survivin mRNA and protein expression. The underlying mechanism involves the disruption of Stat3 or NF-κB binding of their DNA sites at the survivin promoter by SF002-96-1 in Colo320 cells 75. WM-127 suppresses survivin protein level and cell viability. Mechanistic studies showed that WM-127 inhibited the activity of the Survivin/β-catenin pathway and induced the expression of Bax 76. Our results showed that the natural compound dioscin also decreased survivin gene expression. However, more experiments need to be performed to evaluate the anticancer activity of dioscin both *in vitro* and *in vivo*.

The mechanisms of anti-apoptotic activity of survivin have not been fully elucidated. Survivin most probably interferes in the downstream steps of the mitochondrial-apoptotic pathway, such as blocking the activation of caspase 9 by antagonizing apoptosome formation 77. It may also inhibit apoptotic effector caspase 3 directly 78. Additionally, survivin may suppress the activation of caspases indirectly through its interaction with Smac/DIABLO and subsequently antagonize the activity of Smac/DIABLO, which acts as proapoptotic protein by the participation in the formation of apoptosome and activation of caspase 9 79, 80. Our results indicated that dioscin-induced apoptosis of OSCC cells accompanied with survivin downregulation and the cleavage of procaspase 3 (Fig. 2A). Further studies would be required to unravel the precise proapoptotic mechanisms by which dioscin-downregulated survivin induces the apoptosis of OSCC cells.

In summary, we demonstrated in this study that dioscin inhibits survivin expression by interrupting EGFR binding to the ATRSs at the survivin promoter, eventually promoting survivin-mediated cell apoptosis. Thus, targeting this oncoprotein for downregulation might be a promising strategy for OSCC therapy.

## Figures and Tables

**Table 1:** Primers for amplification of the Survivin promoter by ChIP-PCR.

Primer	Sense sequence (5'to3')	Antisense sequence (5'to3')	Size (bp)
1	AGGGGTAAGAGAGGGAGAGG	AGGCTGGTCTCAAACTCCTG	247
2	CAGGAGACAGAGAGAGAGCG	CCGGCCCGATGTCAATTTAA	199
3	TGAGCTGAGATCATGCCACT	CTTATTAGCCCTCCAGCCCC	222
4	GAAGTGAGTGGATGTGATGCC	AGGGCAAACTCCTCCTTTCC	151
5	CCATCCCTCCCCTGTTCATT	GTGGTGCATGCCTGTAATCC	241
6	GATGTCTGCTGCACTCCATC	AGTGAGCTGAGATTGTGCCA	179
7	CACCACGCCCAGCTAATTTT	ACTGCTTTCAAAGAACGCGT	172
8	GGGTTCAAGCGATTCTCCTG	CTTTCAAAGAACGCGTGCAG	226
9	CGCCTCTACTCCCAGAAGG	GTAGAGATGCGGTGGTCCTT	193
10	GACTACAACTCCCGGCACA	ATGCGGTGGTCCTTGAGAAA	219

## References

[1] Troiano G, Guida A, Aquino G, Botti G, Losito NS, Papagerakis S (2018). Integrative Histologic and Bioinformatics Analysis of BIRC5/Survivin Expression in Oral Squamous Cell Carcinoma. Int J Mol Sci.

[2] Sarode GS, Sarode SC, Maniyar N, Anand R, Patil S (2018). Oral cancer databases: A comprehensive review. JOURNAL OF ORAL PATHOLOGY & MEDICINE.

[3] Ghantous Y, Abu Elnaaj I (2017). [GLOBAL INCIDENCE AND RISK FACTORS OF ORAL CANCER]. Harefuah.

[4] Herrero R, Castellsague X, Pawlita M, Lissowska J, Kee F, Balaram P (2003). Human papillomavirus and oral cancer: The international agency for research on cancer multicenter study. JNCI-JOURNAL OF THE NATIONAL CANCER INSTITUTE.

[5] Mody MD, Rocco JW, Yom SS, Haddad RI, Saba NF (2021). Head and neck cancer. LANCET.

[6] Lau A, Li K-Y, Yang W-f, Su Y-X (2016). Induction chemotherapy for squamous cell carcinomas of the oral cavity: A cumulative meta-analysis. ORAL ONCOLOGY.

[7] Li M, Gao F, Yu X, Zhao Q, Zhou L, Liu W (2020). Promotion of ubiquitination-dependent survivin destruction contributes to xanthohumol-mediated tumor suppression and overcomes radioresistance in human oral squamous cell carcinoma. JOURNAL OF EXPERIMENTAL & CLINICAL CANCER RESEARCH.

[8] Li M, Liu H, Zhao Q, Han S, Zhou L, Liu W (2021). Targeting Aurora B kinase with Tanshinone IIA suppresses tumor growth and overcomes radioresistance. Cell Death Dis.

[9] Li XY, Gao F, Wang XC, Liu LL, Gan Y, Han SZ (2023). Curcumol Exerts Antitumor Effect via Inhibiting EGFR-Akt-Mcl-1 Signaling. Am J Chin Med.

[10] Yu X, Gao F, Li W, Zhou L, Liu W, Li M (2020). Formononetin inhibits tumor growth by suppression of EGFR-Akt-Mcl-1 axis in non-small cell lung cancer. J Exp Clin Cancer Res.

[11] Sophia J, Kowshik J, Dwivedi A, Bhutia SK, Manavathi B, Mishra R (2018). Nimbolide, a neem limonoid inhibits cytoprotective autophagy to activate apoptosis via modulation of the PI3K/Akt/GSK-3β signalling pathway in oral cancer. Cell Death Dis.

[12] Zhou L, Yu X, Li M, Gong G, Liu W, Li T (2020). Cdh1-mediated Skp2 degradation by dioscin reprogrammes aerobic glycolysis and inhibits colorectal cancer cells growth. EBIOMEDICINE.

[13] Xiao Y, Liu F, Zhu X, Li S, Meng L, Jiang N (2023). Dioscin integrates regulation of monosaturated fatty acid metabolism to extend the life span through XBP-1/SBP-1 dependent manner. iScience.

[14] Wang D, Wang X (2022). Diosgenin and Its Analogs: Potential Protective Agents Against Atherosclerosis. Drug Des Devel Ther.

[15] Bandopadhyay S, Anand U, Gadekar VS, Jha NK, Gupta PK, Behl T (2022). Dioscin: A review on pharmacological properties and therapeutic values. Biofactors.

[16] Qu PR, Jiang ZL, Song PP, Liu LC, Xiang M, Wang J (2022). Saponins and their derivatives: Potential candidates to alleviate anthracycline-induced cardiotoxicity and multidrug resistance. Pharmacol Res.

[17] Shang Q, Zhu L, Shang W, Zeng J, Qi Y (2022). Dioscin exhibits protective effects on *in vivo* and *in vitro* asthma models via suppressing TGF-β1/Smad2/3 and AKT pathways. J Biochem Mol Toxicol.

[18] Li R, Qin J, Wang Z, Lv F, Guo J, Zhu H (2023). Dioscin reduced chemoresistance for colon cancer and analysis of sensitizing targets. Biochem Biophys Res Commun.

[19] Xun J, Zhou S, Lv Z, Wang B, Luo H, Zhang L (2023). Dioscin modulates macrophages polarization and MDSCs differentiation to inhibit tumorigenesis of colitis-associated colorectal cancer. Int Immunopharmacol.

[20] Hsieh M-J, Tsai T-L, Hsieh Y-S, Wang C-J, Chiou H-L (2013). Dioscin-induced autophagy mitigates cell apoptosis through modulation of PI3K/Akt and ERK and JNK signaling pathways in human lung cancer cell lines. ARCHIVES OF TOXICOLOGY.

[21] He S, Yang J, Hong S, Huang H, Zhu Q, Ye L (2020). Dioscin Promotes Prostate Cancer Cell Apoptosis and Inhibits Cell Invasion by Increasing SHP1 Phosphorylation and Suppressing the Subsequent MAPK Signaling Pathway. FRONTIERS IN PHARMACOLOGY.

[22] Zhao X, Xu L, Zheng L, Yin L, Qi Y, Han X (2016). Potent effects of dioscin against gastric cancer *in vitro* and *in vivo*. PHYTOMEDICINE.

[23] Guo X, Ding X (2018). Dioscin suppresses the viability of ovarian cancer cells by regulating the VEGFR2 and PI3K/AKT/MAPK signaling pathways. ONCOLOGY LETTERS.

[24] Si L, Zheng L, Xu L, Yin L, Han X, Qi Y (2016). Dioscin suppresses human laryngeal cancer cells growth via induction of cell-cycle arrest and MAPK-mediated mitochondrial-derived apoptosis and inhibition of tumor invasion. EUROPEAN JOURNAL OF PHARMACOLOGY.

[25] Vlčková K, Ondrušová L, Vachtenheim J, Réda J, Dundr P, Zadinová M (2016). Survivin, a novel target of the Hedgehog/GLI signaling pathway in human tumor cells. Cell Death Dis.

[26] Zhang L, Zhang W, Wang YF, Liu B, Zhang WF, Zhao YF (2015). Dual induction of apoptotic and autophagic cell death by targeting survivin in head neck squamous cell carcinoma. CELL DEATH & DISEASE.

[27] Yang C-T, Li J-M, Li L-F, Ko Y-S, Chen J-T (2018). Stomatin-like protein 2 regulates survivin expression in non-small cell lung cancer cells through beta-catenin signaling pathway. CELL DEATH & DISEASE.

[28] Charette N, De Saeger C, Horsmans Y, Leclercq I, Starkel P (2013). Salirasib sensitizes hepatocarcinoma cells to TRAIL-induced apoptosis through DR5 and survivin-dependent mechanisms. CELL DEATH & DISEASE.

[29] Garlapati C, Joshi S, Bhattarai S, Krishnamurthy J, Turaga RC, Nguyen T (2023). PLK1 and AURKB phosphorylate survivin differentially to affect proliferation in racially distinct triple-negative breast cancer. CELL DEATH & DISEASE.

[30] Singh N, Krishnakumar S, Kanwar RK, Cheung CHA, Kanwar JR (2015). Clinical aspects for survivin: a crucial molecule for targeting drug-resistant cancers. DRUG DISCOVERY TODAY.

[31] Li M, Gao F, Li X, Gan Y, Han S, Yu X (2022). Stabilization of MCL-1 by E3 ligase TRAF4 confers radioresistance. CELL DEATH & DISEASE.

[32] Dong X, Li X, Gan Y, Ding J, Wei B, Zhou L (2023). TRAF4-mediated ubiquitination-dependent activation of JNK/Bcl-xL drives radioresistance. Cell Death Dis.

[33] Li M, Gao F, Zhao Q, Zuo H, Liu W, Li W (2020). Tanshinone IIA inhibits oral squamous cell carcinoma via reducing Akt-c-Myc signaling-mediated aerobic glycolysis. CELL DEATH & DISEASE.

[34] Yu X, Wang R, Zhang Y, Zhou L, Wang W, Liu H (2019). Skp2-mediated ubiquitination and mitochondrial localization of Akt drive tumor growth and chemoresistance to cisplatin. Oncogene.

[35] Zhu H-X, Zhang G, Wang Y-H, Zhou C-Q, Bai J-F, Xu N-Z (2004). [Indomethacin induces apoptosis through inhibition of survivin regulated by beta-catenin/TCF4 in human colorectal cancer cells]. Ai zheng = Aizheng = Chinese journal of cancer.

[36] Li W, Yu X, Xia Z, Yu X, Xie L, Ma X (2018). Repression of Noxa by Bmi1 contributes to deguelin-induced apoptosis in non-small cell lung cancer cells. JOURNAL OF CELLULAR AND MOLECULAR MEDICINE.

[37] Wu Z, Han X, Tan G, Zhu Q, Chen H, Xia Y (2020). Dioscin Inhibited Glycolysis and Induced Cell Apoptosis in Colorectal Cancer via Promoting c-myc Ubiquitination and Subsequent Hexokinase-2 Suppression. Onco Targets Ther.

[38] Song X, Wang Z, Liang H, Zhang W, Ye Y, Li H (2017). Dioscin Induces Gallbladder Cancer Apoptosis by Inhibiting ROS-Mediated PI3K/AKT Signalling. Int J Biol Sci.

[39] Gao F, Yu X, Li M, Zhou L, Liu W, Li W (2020). Deguelin suppresses non-small cell lung cancer by inhibiting EGFR signaling and promoting GSK3β/FBW7-mediated Mcl-1 destabilization. Cell Death Dis.

[40] Lin SY, Makino K, Xia WY, Matin A, Wen Y, Kwong KY (2001). Nuclear localization of EGF receptor and its potential new role as a transcription factor. NATURE CELL BIOLOGY.

[41] Hung L-Y, Tseng JT, Lee Y-C, Xia W, Wang Y-N, Wu M-L (2008). Nuclear epidermal growth factor receptor (EGFR) interacts with signal transducer and activator of transcription 5 (STAT5) in activating Aurora-A gene expression. NUCLEIC ACIDS RESEARCH.

[42] Troiano G, Guida A, Aquino G, Botti G, Losito NS, Papagerakis S (2018). Integrative Histologic and Bioinformatics Analysis of BIRC5/Survivin Expression in Oral Squamous Cell Carcinoma. INTERNATIONAL JOURNAL OF MOLECULAR SCIENCES.

[43] Xie S, Xu H, Shan X, Liu B, Wang K, Cai Z (2015). Clinicopathological and Prognostic Significance of Survivin Expression in Patients with Oral Squamous Cell Carcinoma: Evidence from a Meta-Analysis. PLOS ONE.

[44] Yu X, Zhang Y, Cavazos D, Ma X, Zhao Z, Du L (2018). miR-195 targets cyclin D3 and survivin to modulate the tumorigenesis of non-small cell lung cancer. CELL DEATH & DISEASE.

[45] Jiang X, Finucane HK, Schumacher FR, Schmit SL, Tyrer JP, Han Y (2019). Shared heritability and functional enrichment across six solid cancers. NATURE COMMUNICATIONS.

[46] Nantajit D, Presta L, Sauter T, Tavassoli M (2022). EGFR-induced suppression of HPV E6/E7 is mediated by microRNA-9-5p silencing of BRD4 protein in HPV-positive head and neck squamous cell carcinoma. Cell Death Dis.

[47] Chandarana SP, Lee JS, Chanowski EJP, Sacco AG, Bradford CR, Wolf GT (2013). Prevalence and predictive role of p16 and epidermal growth factor receptor in surgically treated oropharyngeal and oral cavity cancer. HEAD AND NECK-JOURNAL FOR THE SCIENCES AND SPECIALTIES OF THE HEAD AND NECK.

[48] Bundela S, Sharma A, Bisen PS (2014). Potential Therapeutic Targets for Oral Cancer: ADM, TP53, EGFR, LYN, CTLA4, SKIL, CTGF, CD70. PLOS ONE.

[49] William WN Jr, Papadimitrakopoulou V, Lee JJ, Mao L, Cohen EEW, Lin HY (2016). Erlotinib and the Risk of Oral Cancer The Erlotinib Prevention of Oral Cancer (EPOC) Randomized Clinical Trial. JAMA ONCOLOGY.

[50] Nakamura H, Tamaki S, Yagyuu T, Yamakawa N, Hatake K, Kirita T (2019). Relationship Between EGFR Expression in Oral Cancer Cell Lines and Cetuximab Antibody-dependent Cell-mediated Cytotoxicity. ANTICANCER RESEARCH.

[51] Wells A, Marti U (2002). Signalling shortcuts: cell-surface receptors in the nucleus?. NATURE REVIEWS MOLECULAR CELL BIOLOGY.

[52] Offterdinger M, Schofer C, Weipoltshammer K, Grunt TW (2002). c-erbB-3: a nuclear protein in mammary epithelial cells. JOURNAL OF CELL BIOLOGY.

[53] Lo HW, Hsu SC, Ali-Seyed M, Gunduz M, Xia WY, Wei YK (2005). Nuclear interaction of EGFR and STAT3 in the activation of the iNOS/NO pathway. CANCER CELL.

[54] Lo H-W, Cao X, Zhu H, Ali-Osman F (2010). Cyclooxygenase-2 Is a Novel Transcriptional Target of the Nuclear EGFR-STAT3 and EGFRvIII-STAT3 Signaling Axes. MOLECULAR CANCER RESEARCH.

[55] Kuo H-Y, Huang Y-S, Tseng C-H, Chen Y-C, Chang Y-W, Shih H-M (2014). PML represses lung cancer metastasis by suppressing the nuclear EGFR-mediated transcriptional activation of MMP2. CELL CYCLE.

[56] Cordero JB, Cozzolino M, Lu Y, Vidal M, Slatopolsky E, Stahl PD (2002). 1,25-dihydroxyvitamin D down-regulates cell membrane growth- and nuclear growth-promoting signals by the epidermal growth factor receptor. JOURNAL OF BIOLOGICAL CHEMISTRY.

[57] Peng H, Moffett J, Myers J, Fang XH, Stachowiak EK, Maher P (2001). Novel nuclear signaling pathway mediates activation of fibroblast growth factor-2 gene by type 1 and type 2 angiotensin II receptors. MOLECULAR BIOLOGY OF THE CELL.

[58] Papanikolaou V, Iliopoulos D, Dimou I, Dubos S, Kappas C, Kitsiou-Tzeli S (2011). Survivin regulation by HER2 through NF-kappa B and c-myc in irradiated breast cancer cells. JOURNAL OF CELLULAR AND MOLECULAR MEDICINE.

[59] Yang J, Song K, Krebs TL, Jackson MW, Danielpour D (2008). Rb/E2F4 and Smad2/3 link survivin to TGF-beta-induced apoptosis and tumor progression. ONCOGENE.

[60] Zhang Y, Chen H-x, Zhou S-y, Wang S-x, Zheng K, Xu D-d (2015). Sp1 and c-Myc modulate drug resistance of leukemia stem cells by regulating survivin expression through the ERK-MSK MAPK signaling pathway. MOLECULAR CANCER.

[61] Vaziri SAJ, Hill J, Chikamori K, Grabowski DR, Takigawa N, Chawla-Sarkar M (2005). Sensitization of DNA damage-induced apoptosis by the proteasome inhibitor PS-341 is p53 dependent and involves target proteins 14-3-3 sigma and survivin. MOLECULAR CANCER THERAPEUTICS.

[62] Wagner M, Schmelz K, Doerken B, Tamm I (2008). Transcriptional regulation of human survivin by early growth response (Egr)-1 transcription factor. INTERNATIONAL JOURNAL OF CANCER.

[63] Arora V, Cheung HH, Plenchette S, Micali OC, Liston P, Korneluk RG (2007). Degradation of survivin by the x-linked inhibitor of apoptosis (XIAP)-XAF1 complex. JOURNAL OF BIOLOGICAL CHEMISTRY.

[64] Li Z, Pei X-H, Yan J, Yan F, Cappell KM, Whitehurst AW (2014). CUL9 Mediates the Functions of the 3M Complex and Ubiquitylates Survivin to Maintain Genome Integrity. MOLECULAR CELL.

[65] Liu Y, Lear T, Iannone O, Shiva S, Corey C, Rajbhandari S (2015). The Proapoptotic F-box Protein Fbxl7 Regulates Mitochondrial Function by Mediating the Ubiquitylation and Proteasomal Degradation of Survivin. JOURNAL OF BIOLOGICAL CHEMISTRY.

[66] Woo SM, Seo SU, Kubatka P, Min K-J, Kwon TK (2019). Honokiol Enhances TRAIL-Mediated Apoptosis through STAMBPL1-Induced Survivin and c-FLIP Degradation. BIOMOLECULES.

[67] Woo SM, Kim S, Seo SU, Kim S, Park J-W, Kim G (2022). Inhibition of USP1 enhances anticancer drugs-induced cancer cell death through downregulation of survivin and miR-216a-5p-mediated upregulation of DR5. CELL DEATH & DISEASE.

[68] Chandrasekaran AP, Tyagi A, Poondla N, Sarodaya N, Karapurkar JK, Kaushal K (2022). Dual role of deubiquitinating enzyme USP19 regulates mitotic progression and tumorigenesis by stabilizing survivin. MOLECULAR THERAPY.

[69] Yun S, Kim WK, Kwon Y, Jang M, Bauer S, Kim H (2018). Survivin is a novel transcription regulator of KIT and is downregulated by miRNA-494 in gastrointestinal stromal tumors. INTERNATIONAL JOURNAL OF CANCER.

[70] Diakos C, Zhong S, Xiao Y, Zhou M, Vasconcelos GM, Krapf G (2010). TEL-AML1 regulation of survivin and apoptosis via miRNA-494 and miRNA-320a. BLOOD.

[71] Lyu H, Huang J, He Z, Liu B (2018). Epigenetic mechanism of survivin dysregulation in human cancer. SCIENCE CHINA-LIFE SCIENCES.

[72] Johnson B, Zhuang L, Rath EM, Yuen ML, Cheng NC, Shi H (2022). Exploring MicroRNA and Exosome Involvement in Malignant Pleural Mesothelioma Drug Response. Cancers (Basel).

[73] Voges Y, Michaelis M, Rothweiler F, Schaller T, Schneider C, Politt K (2016). Effects of YM155 on survivin levels and viability in neuroblastoma cells with acquired drug resistance. CELL DEATH & DISEASE.

[74] Ling X, Cao S, Cheng Q, Keefe JT, Rustum YM, Li F (2012). A Novel Small Molecule FL118 That Selectively Inhibits Survivin, Mcl-1, XIAP and cIAP2 in a p53-Independent Manner, Shows Superior Antitumor Activity. PLOS ONE.

[75] Felix S, Sandjo LP, Opatz T, Erkel G (2013). SF002-96-1, a new drimane sesquiterpene lactone from an Aspergillus species, inhibits survivin expression. BEILSTEIN JOURNAL OF ORGANIC CHEMISTRY.

[76] Yin H, Que R, Liu C, Ji W, Sun B, Lin X (2018). Survivin-targeted drug screening platform identifies a matrine derivative WM-127 as a potential therapeutics against hepatocellular carcinoma. CANCER LETTERS.

[77] Tamm I, Wang Y, Sausville E, Scudiero DA, Vigna N, Oltersdorf T (1998). IAP-family protein survivin inhibits caspase activity and apoptosis induced by Fas (CD95), Bax, caspases, and anticancer drugs. Cancer research.

[78] Khan Z, Khan AA, Yadav H, Prasad GBKS, Bisen PS (2017). Survivin, a molecular target for therapeutic interventions in squamous cell carcinoma. CELLULAR & MOLECULAR BIOLOGY LETTERS.

[79] Khan Z, Bisen PS (2013). Oncoapoptotic signaling and deregulated target genes in cancers: Special reference to oral cancer. BIOCHIMICA ET BIOPHYSICA ACTA-REVIEWS ON CANCER.

[80] Udoh U-AS, Banerjee M, Rajan PK, Sanabria JD, Smith G, Schade M (2022). Tumor-Suppressor Role of the alpha 1-Na/K-ATPase Signalosome in NASH Related Hepatocellular Carcinoma. INTERNATIONAL JOURNAL OF MOLECULAR SCIENCES.

